# From Crystals to Disordered Crystals: A Hidden Order-Disorder Transition

**DOI:** 10.1038/srep15378

**Published:** 2015-10-20

**Authors:** Hua Tong, Peng Tan, Ning Xu

**Affiliations:** 1 CAS Key Laboratory of Soft Matter Chemistry, Hefei National Laboratory for Physical Sciences at the Microscale, and Department of Physics, University of Science and Technology of China, Hefei 230026, People’s Republic of China; 2 State Key Laboratory of Surface Physics and Department of Physics, Fudan University, Shanghai 200433, People’s Republic of China

## Abstract

To distinguish between order and disorder is of fundamental importance to understanding solids. It becomes more significant with recent observations that solids with high structural order can behave like disordered solids, while properties of disordered solids can approach crystals under certain circumstance. It is then imperative to understand when and how disorder takes effect to deviate the properties of a solid from crystals and what the correct factors are to control the behaviours of solids. Here we answer these questions by reporting the finding of a hidden order-disorder transition from crystals to disordered crystals for static packings of frictionless spheres. While the geometric indicators are mostly blind to the transition, disordered crystals already exhibit properties apart from crystals. The transition approaches the close packing of hard spheres, giving rise to the singularity of the close packing point. We evidence that both the transition and properties of disordered crystals are jointly determined by the structural order and density. Near the transition, the elastic moduli and coordination number of disordered crystals show particular pressure dependence distinct from known behaviours of both crystals and jammed solids. The discovery of the transition therefore reveals some unknown aspects of solids.

Order and disorder constitute two fundamental themes in condensed matter physics and materials science. An integrated understanding of materials over the entire spectrum of disorder requires organizing principles from both sides, and in between. Perfect crystals, the epitome of order, provide an important starting point for understanding properties of solids, which lie in the heart of solid state physics^1^. In contrast, materials such as glasses and granular assemblies are highly disordered^2^,^3^,^4^,^5^,^6^,^7^. These amorphous materials exhibit a set of universal properties distinct from their crystalline counterparts, which are the research focus of soft condensed matter physics^2^,^3^,^4^,^5^,^6^,^7^,^8^. Expectedly, a crystal can evolve away from the perfect crystalline order and eventually develop into an amorphous state when disorder is introduced^8^,^9^. While intensive efforts have been invested into properties of amorphous solids, the characterization of the intermediate regime between crystals and amorphous solids, especially the crossover between the physics of crystals and that of disordered solids, has not been carefully tackled, leaving the boundary between the two extremes of order and disorder vague.

Intuitively, one may distinguish disordered solids from crystals based on the structural order and simply classify solids with high structural order to crystals. This has been proven infeasible because sometimes solids with extremely high structural order could exhibit features of disordered solids^10^,^11^; whereas those with considerably low structural order may respond more like a crystal^11^. Moreover, a recent experiment showed that a glass could exhibit low-temperature thermodynamic properties more like polycrystals when being compressed to high pressures^12^. It was then claimed that lower density, rather than disorder, might be the reason why glasses behave differently from crystals. Although surprising, this study at least indicates that in addition to the structural order there exist other possible controlling parameters of the manifestation of disordered solids, while the density is a candidate. However, how the density works together with the structural order to determine properties of disordered solids is still an open question.

Bearing in mind the questions mentioned above, we numerically investigate the evolution from perfect crystals to disordered solids, by using the particle-size polydispersity as the control parameter. In the similar framework, previous simulations have shown that the system undergoes the structural amorphisation toward an amorphous solid state when the polydispersity increases to a sufficiently large value^13^,^14^. Our characterizations of the similar amorphisation transition are described in Section I of PART ONE of the Supplementary Information.

Here we focus on another hidden order-disorder transition at a rather small polydispersity from crystals to *disordered crystals*, namely solids with extremely high crystalline order in structure but mechanical and vibrational properties resembling disordered solids. While the bond orientational and translational orders^8^,^9^,^15^,^16^ are insensitive to this transition at all, multiple quantities undergo a sudden change. We propose the spatial fluctuation of the coordination number *δz* as the order parameter to characterize this unusual order-disorder transition. It turns out that the critical polydispersity of the transition *η*_*c*_ is scaled linearly with the packing-fraction distance from the close packing of hard spheres, *ϕ* − *ϕ*_cp_. Therefore, the close packing behaves like a singular point where infinitesimally small polydispersity turns the crystal into a disordered crystal^10^. In the following, we will show that the significance of this transition is manifested by unknown physics that it brings about, which unveils important aspects of solids and answers the questions raised above. A unified phase diagram of solids over the entire spectrum of disorder is conveyed in Fig. 1a with both the transitions from crystals to disordered crystals and from disordered crystals to amorphous solids. Here we denote disordered solids with strong structural amorphisation as amorphous solids to distinguish them from disordered crystals.

## Results

### Model

**Figure 1:**
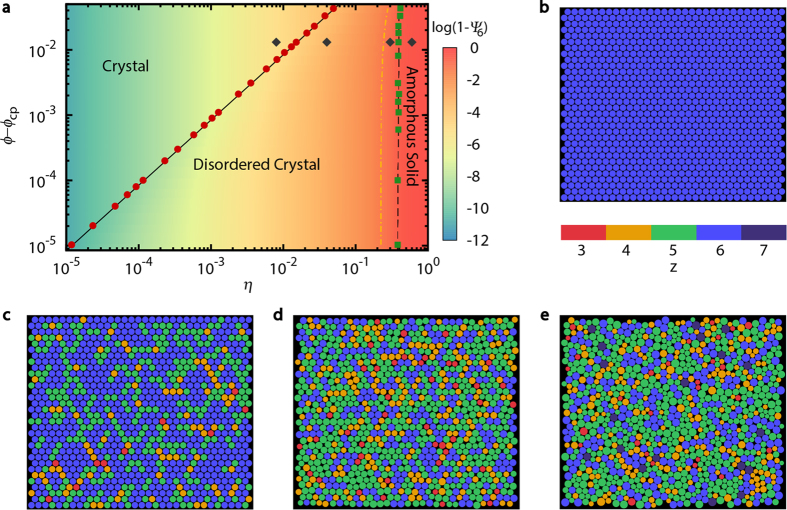
Phase diagram and evolution from crystals to amorphous solids. (**a**) Phase diagram with two order-disorder transitions in the parametric space of the particle-size polydispersity *η* and packing fraction distance from the close packing of hard spheres *ϕ* − *ϕ*_cp_. With increasing *η* at fixed *ϕ*, the system starting from a perfect triangular crystal undergoes a transition at *η*_*c*_ toward a disordered crystal state, which is labeled by the circles with the linear fit 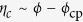 (solid line). *η*_*c*_ = 0 when *ϕ* = *ϕ*_cp_, indicating that the close packing point is singular. Across this transition, the geometric structure maintains an extremely high crystalline order, whereas mechanical and vibrational properties become more like disordered solids. The transition labeled by the squares signals the structural amorphisation from disordered crystals to amorphous solids. The dashed line is to guide the eye. The colour contour shows 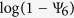 with 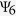 the bond orientational order. The dot-dashed line marks 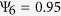, to the left of which all states have a high crystalline order 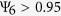. (**b**–**e**) Configurations of a crystal, a disordered crystal, a strongly disordered crystal, and an amorphous solids whose locations in the phase diagram are labeled by the diamonds in (**a**). The colour coding shows the coordination number of each particle. Note that without the colour coding (**b**,**c**) would be undistinguishable with the eyes.

Our model systems consist of frictionless particles interacting via finite range repulsions, which mimic experimental systems like granular and colloidal solids^17^,^18^,^19^. By means of quasistatic modulation of the particle-size polydispersity *η*, we continuously tune a perfect crystal into a disordered solid (see Methods). Since disorder is introduced quasistatically in a spatially random and uniform manner, the strength of the global disorder is well controlled by *η*. As shown in Figs. S1b and S1e of the Supplementary Information, both the bond orientational and translational orders decrease monotonically with increasing *η* and show almost no packing fraction dependence. The bond orientational order is visualized as well by the colour contour in Fig. 1a. We show here results of two-dimensional (2D) packings of disks with harmonic repulsion. We have also performed extensive studies on three-dimensional (3D) packings with harmonic repulsion and 2D packings with Hertzian repulsion and Gaussian particle-size distribution (shown in PART TWO and PART THREE of the Supplementary Information respectively). Essentially the same results are observed.

Figure 1b–e show the evolution from a perfect triangular crystal, through the weakly and strongly disordered crystalline states, to an amorphous solid at fixed packing fraction. The locations of the four states in the (*ϕ*, *η*) plane are labeled by the diamonds in Fig. 1a. The crystal (Fig. 1b) and weakly disordered crystal (Fig. 1c) are undistinguishable with the eyes purely from the geometric structure. However, as illustrated by the colour coding, an underlying order-disorder transition emerges between them. The colour on each particle demonstrates the coordination number, i.e., the number of interacting neighbors.

### Order Parameter

**Figure 2:**
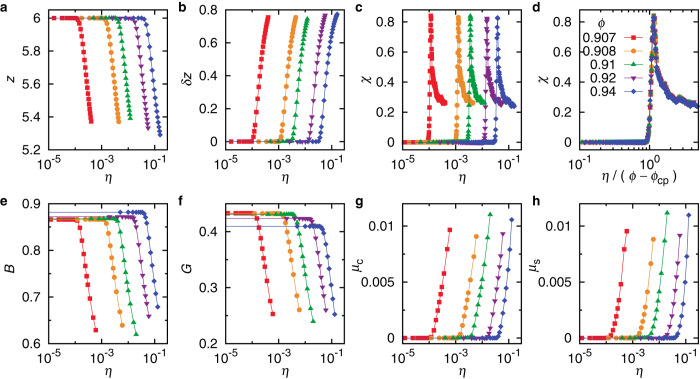
Order parameter and elastic properties across the transition from crystals to disordered crystals. (**a–c**) Polydispersity evolution of the average coordination number *z*, fluctuation of the coordination number *δz* as the order parameter, and susceptibility of the order parameter χ. The critical polydispersity of the transition *η*_*c*_ is determined by the location of the peak in χ(*η*). (**d**) Scaling collapse of all curves in (**c**) when χ is plotted against 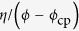. (**e–h**) Polydispersity evolution of the bulk modulus *B*, shear modulus *G*, nonaffinity of the compression deformation *μ*_*c*_, and nonaffinity of the shear deformation *μ*_*s*_. Note that systems at higher *ϕ* and with larger *η* (stronger structural disorder) can exhibit quantitatively similar mechanical properties to those at lower *ϕ* and with smaller *η*.

When a crystal is driven progressively into the disordered crystal state, the contact network distorts continuously with increasing polydispersity and is eventually destroyed by some local contact breaking. Consequently, the average coordination number *z* drops below 6, as shown in Fig. 2a. Rattlers are not included in our calculation of *z*. The contact breaking happens randomly in space, resulting in the spatially heterogeneous disorder, which is one of the most important features of disordered solids^2^,^3^,^20^,^21^,^22^,^23^,^24^. We thus propose the spatial fluctuation of the coordination number, 
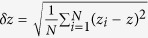
, as the order parameter to characterize the strength of disorder, where *z*_*i*_ is the coordination number of particle *i*. As shown in Fig. 2b, *δz* increases quickly from zero at some polydispersity, signaling the transition from crystals to disordered crystals. Here we focus on the physics around such a transition. When *η* further increases, *z* continues to drop and levels off at a *ϕ*-dependent value larger than the isostatic value, because the systems are far beyond the random close packing. Rattlers also emerge at certain *η* much higher than the critical value at the transition, which are thus irrelevant to the transition from crystals to disordered crystals.

In order to unambiguously determine the transition point, we calculate the order parameter susceptibility 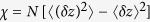, where 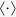 denotes the average over 1000 distinct realizations under the same macroscopic conditions. The susceptibility method has been proven to be superior in locating the phase transition point^25^ in studies of phase transitions, e.g., 2D melting^26^,^27^,^28^ and transition from crystals to glasses^29^. As shown in Fig. 2c, there is a peak in 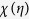, whose location *η*_*c*_ is defined here as the transition point from crystals to disordered crystals. As illustrated by the circles in Fig. 1a, 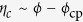, where 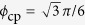 is the packing fraction of close-packed hard spheres. When 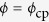, *η*_*c*_ = 0. Infinitesimally small polydispersity will trigger the transition, so the close packing point is singular.

Seen from Fig. 2c, 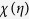 at different packing fractions look alike. When *χ* is plotted against 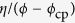 as in Fig. 2d, interestingly, all curves collapse nicely onto the same master curve, suggesting that 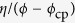 be a more meaningful parameter in control of the transition from crystals to disordered crystals.

We stress that the occurrence of the transition is non-trivial. It is not simply caused by the breaking of a few isolated contacts, which just slightly perturbs the properties of crystals. It is the accumulation of heterogeneous contact breakings that leads to the cooperative effects at the transition and qualitative changes of the properties of solids.

### Elastic moduli and nonaffinity

In Fig. 2e–h, we show the *η* evolution of typical properties concerned in the characterization of disordered solids, including the elastic moduli and nonaffinity upon deformation. All quantities undergo remarkable changes across *η*_*c*_. In the crystal regime, both the bulk modulus *B* and shear modulus *G* remain mostly constant in *η*. Meanwhile, both the compression and shear deformations are affine with the corresponding nonaffinity 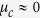 and 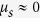 (see Methods for the definition of *μ*_*c*_ and *μ*_*s*_). When 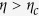, *B* and *G* decrease, while *μ*_*c*_ and *μ*_*s*_ increase, all at once. These sudden changes strongly verify the validity and robustness of the crystal-disordered crystal transition.

Figure 2e–h also demonstrate that disordered crystals at higher packing fractions and larger polydispersities (stronger structural disorder) can have quantitatively similar mechanical properties to those at lower packing fractions and smaller polydispersities (weaker structural disorder). In addition to the transition at *η*_*c*_, properties of disordered crystals seem to be jointly determined by the structural order and the packing fraction as well in the form of 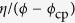. This provides us with some clues to understand the puzzle why compressed glasses can behave like polycrystals^12^. When the density of a glass is increased, the ratio of the structural disorder and the density is smaller and approaches the value of polycrystals, which pushes properties of the glass closer to polycrystals. Therefore, it is the interplay between the structural order and the density that determines the performance of a solid. To claim either of them to be deterministic is partial.

### Boson peak

**Figure 3:**
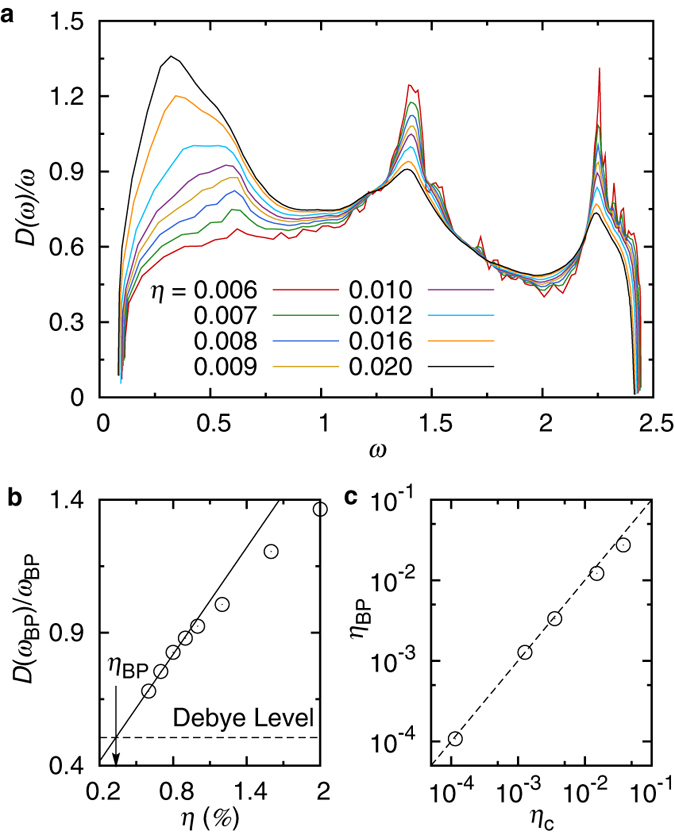
Formation of the boson peak in disordered crystals. (**a**) Reduced density of vibrational states 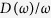 at *ϕ* = 0.91 and different polydispersities. From the left to the right, the three peaks are respectively the boson peak and two van Hove singularities. Note that the boson peak is more pronounced with increasing polydispersity *η*. (**b**) Polydispersity evolution of the strength of the boson peak 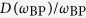 from (**a**). The solid line is a linear fit to the low *η* data. It hits the Debye level labeled by the horizontal dashed line at *η*_BP_. (**c**) Correlation between *η*_BP_ and *η*_*c*_. The data points correspond to five packing fractions of *ϕ* = 0.907, 0.908, 0.91, 0.92, and 0.94 in the ascendent order of *η*_*c*_. The dashed line shows *η*_BP_ = *η*_*c*_. The good agreement between *η*_*c*_ and *η*_BP_ suggests that the boson peak emerges as a result of the transition from crystals to disordered crystals.

In addition to mechanical properties, Fig. 3 manifest further the importance of disorder to disordered crystals from vibrational properties. One of the most special vibrational features of disordered solids is the boson peak, i.e., the peak in 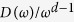 with 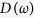 the density of vibrational states, and *ω* the frequency^2^,^3^,^30^,^31^,^32^,^33^,^34^. For perfect crystals, the low-frequency *D*(*ω*) obeys the Debye law: 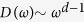. The emergence of the boson peak indicates that excess number of low-frequency modes beyond Debye’s prediction are induced by disorder. It is believed that the boson peak contributes to a variety of low-temperature anomalies of disordered solids^2^,^3^,^30^,^31^. Figure 3a shows the reduced density of states 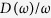 for disordered crystals at *ϕ* = 0.91. To smooth out the planewave-like peaks due to the finite size effect, we average *D*(*ω*) calculated at different system sizes ranging from *N* = 256 to 1024^32^. With increasing *η*, the boson peak (the first peak at low frequencies) gradually rises and moves to lower frequencies, which is consistent with the argument that the boson peak is correlated with the structural disorder^31^,^32^,^33^. There are also two other peaks of van Hove singularities in the intermediate and high frequency regimes, whose presence indicates that the solids are still pretty crystalline in structure and possess hybridized characters of crystals and disordered solids.

By plotting 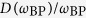 against *η* in Fig. 3b with *ω*_BP_ the boson peak frequency, we estimate below what value of *η* the boson peak disappears. Owing to the very strong finite-size effect close to *η*_*c*_ (see Fig. S4 of the Supplementary Information for an alternate characterization of the mode evolution), we are not able to obtain smooth enough *D*(*ω*) to resolve the boson peak for certain. By extrapolating the roughly linear part of the low *η* data, we find that 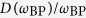 hits the Debye level at 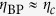. As shown in Fig. 3c, 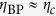 over a wide range of packing fractions from *ϕ* = 0.907 to 0.94. The formation of the boson peak is thus another evidence to distinguish disordered crystals from crystals.

### New physics other than those of crystals and jamming

Recently, it was proposed that solids spanning the entire spectrum of disorder could be described by either the physics of jamming or the physics of crystals^11^. Specifically, for jammed packings of frictionless spheres, both the excess coordination number 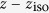 and the ratio of the shear modulus to the bulk modulus *G*/*B* are scaled well with the pressure *P*^35^,^36^,^37^,^38^: 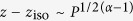 and 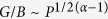 with 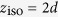 the isostatic value, *d* the dimension of space, and *α* the exponent of the inter-particle potential (see Methods); while for crystals both *z* and *G*/*B* are independent of the pressure. With a more accurate control of structural order, we check how the two types of physics evolve to each other across the crystal-disordered crystal transition.

**Figure 4:**
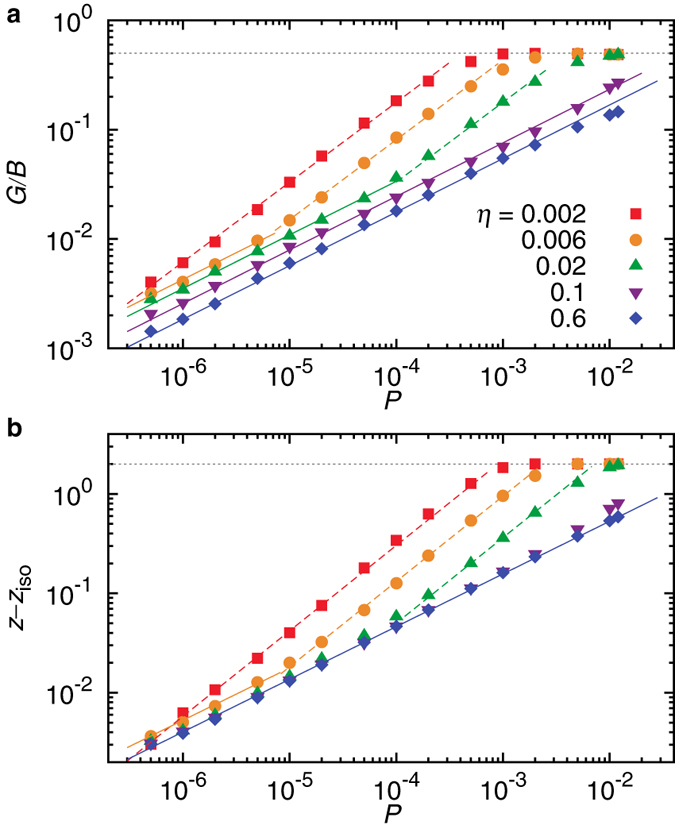
Scaling behaviors of the elastic moduli and coordination number. (**a,b**) Pressure evolution of the ratio of the shear modulus to the bulk modulus *G*/*B* and excess coordination number *z* − *z*_iso_. The horizontal dotted lines show the crystal behavior. The solid (dashed) lines are power-law fits to the data: 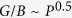 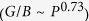 and 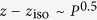 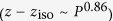. Both *G*/*B* and 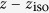 are independent of *P* in the crystal regime and recover the typical scalings of jammed solids away from the transition from crystals to disordered crystals. Close to the transition, a third scaling behavior exists between those of crystalline and jammed packings.

We start from a static packing at *ϕ* = 0.92 and quasistatically decrease the packing fraction at fixed polydispersity to lower the pressure. Figure 4 shows the pressure dependence of *G*/*B* and 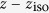 at different *η*, spanning both sides of the transition from crystals to disordered crystals and with lines of amorphous solids at *η* = 0.6 as a reference. When the polydispersity is large, the jamming scalings are recovered over the whole range of pressures studied. When the polydispersity is small, there is a clear transition from the crystal scalings with pressure independent *G*/*B* and 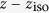 at large pressures to the low pressure scalings disobeying both the physics of crystals and the physics of jamming: 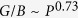 and 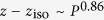. This transition is right at the transition from crystals to disordered crystals. At intermediate polydispersities, the three types of scalings are all present, with the newly reported scalings sitting between those of crystals and jamming. Therefore, disordered crystals close to the crystal-disordered crystal transition comprise a third family of solids complying with the physics other than those of crystals and jamming, whose origin and underlying physics are interesting issues to explore in follow-up studies.

## Discussion

The finding and characterization of the transition from crystals to disordered crystals reveals some unknown features: (i) The close packing point is singular in terms of the transition, implying that it is the only point satisfying the physics of crystals for rigid packings of hard spheres, (ii) the structural order and density interplay to determine the transition and properties of disordered solids, and (iii) disordered crystals near the transition exhibit unique pressure scalings apart from the physics of crystals and the physics of jamming. Here we move a step forward to manifest that our knowledge about solids, even about seemingly crystalline solids, is still rather incomplete.

Follow-up studies, especially to determine the nature of the transition, are necessary to have a deeper understanding of the phenomena reported here. We have performed some finite-size analysis of the transition (see Section V of PART ONE of the Supplementary Information). Our preliminary results indicate that it cannot be easily classified to any well-known type of phase transitions. Another possible explanation of the transition is the percolation of some specific force chains. We have attempted to search for such force chains, but have not found the correct one which undergoes the percolation transition at *η*_*c*_. Intensive studies are required to reveal the nature of the transition, like what have been done to the jamming transition^35^,^36^,^37^,^38^,^39^,^40^,^41^,^42^,^43^,^44^,^45^,^46^,^47^.

One concern that requires further elucidation is how general our major conclusions are. From extensive studies of both 2D and 3D systems with different interactions and different particle size distributions, we think that our major results are robust and are not artifacts of the specific model used. In fact, our observations are highly experimentally relevant. For instance, packings of weakly polydisperse acrylic beads have been shown to posses a highly random mechanical network^19^. Due to the inevitable particle size polydispersity, many seemingly crystalline colloidal solids are actually disordered crystals. Moreover, harmonic and Hertzian repulsions are not limited to the theoretical modeling. For instance, it has been shown that the effective particle interaction of widely studied soft colloidal particles (e.g., poly-N-isopropylacrylamide particles) can be well fitted to these simple repulsions^17^,^18^. For systems with long-range attractions, we also expect similar results, based on the observation that strong repulsive interactions govern the behaviors of the packings while attractions act as perturbations^48^. For more realistic systems such as structural glasses, the formation and manifestation of the structural disorder is complicated, and it is yet unclear how to quantify the disorder in a unified way. However, as an endowed nature of our model, the structural disorder and the polydispersity are positively correlated. So we expect that the physics conveyed by Fig. 1a of the phase diagram is general: If in Fig. 1a the polydispersity is replaced with another quantity characterizing the structural order, we would be able to see the transition from crystals to disordered crystals as well, although the exact manifestation of the transition may rely on the details of the interaction and the style of disorder.

Temperature is another important parameter to include if one tries to generalize our results to thermal systems. Ideally, we can take the temperature as the third axis of the phase diagram (Fig. 1a) to establish a more unified and complete picture. Figure 1a is actually the low-temperature limit. When increasing the temperature, our preliminary results show that the transition from crystals to disordered crystals shifts continuously to lower values of the polydispersity, because the thermal fluctuation is another factor in addition to the polydispersity to induce frustrations (see Section VI of PART ONE of the Supplementary Information for some of our preliminary results). This picture is consistent with previous works where the effects of temperature were studied in monodisperse crystalline solids^49^,^50^,^51^. Therefore, the *T* = 0 case discussed in this paper is not singular from thermal systems, so the physics can be generalized to thermal systems well below the melting temperature. At high temperatures near melting, the solid-liquid transition comes into play and causes more complicated situations^29^,^52^. To settle the temperature effects is undoubtedly worthwhile to do next, which is more relevant to atomic^53^,^54^,^55^ or colloidal systems^29^,^52^,^56^.

## Methods

We start with a perfect crystal, i.e., triangular lattice in two-dimensions (2D) and face-centred cubic lattice in three-dimensions (3D). To ensure perfect crystalline order, we use a rectangular box with the side lengths satisfying 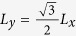 in 2D and 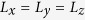 in 3D. Periodic boundary conditions are applied in all directions. All particles have the same mass *m* and interact via the potential


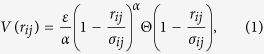


where *r*_*ij*_ is the separation between particles *i* and *j*, *σ*_*ij*_ is the sum of their radii, and 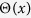 is the Heaviside function. We show here results of 2D systems composed of 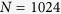 particles with harmonic repulsion (*α* = 2) and present results of 3D harmonic systems in PART TWO and 2D systems with Hertzian repulsion (*α* = 2.5) in PART THREE of the Supplementary Information. The disorder is continuously introduced into the system by tuning the particle-size polydispersity. To do so, we assign each particle a random number *x*_*i*_ uniformly distributed in [−0.5, 0.5] and set the particle diameter *σ*_*I*_ = (1 + *x*_*i*_*η*)*σ* with *η* the polydispersity. We increase *η* from 0 successively by a small step size 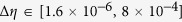, with smaller 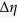 applied to systems closer to the close packing. After each change of *η*, we rescale the average particle radius to make sure that the packing fraction remains unchanged. The system is then relaxed to the local potential energy minimum using the FIRE algorithm^57^. The mass, energy, and length are in units of the particle mass *m*, characteristic energy scale *ε*, and average particle diameter *σ*.

The nonaffinity upon deformation is evaluated by the ratio of the nonaffine and affine particle displacements 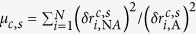, where 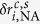 and 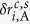 are the nonaffine and affine displacement of particle *i* under a tiny strain in the range of [10^−8^, 10^−6^], with smaller strain applied to systems at lower packing fractions^20^. To calculate the density of vibrational states, we diagonalize the dynamical matrix using ARPACK^58^ to obtain all the normal modes of vibration.

## Additional Information

**How to cite this article**: Tong, H. *et al.* From Crystals to Disordered Crystals: A Hidden Order-Disorder Transition. *Sci. Rep.* **5**, 15378; doi: 10.1038/srep15378 (2015).

## Supplementary Material

Supplementary Information
