# Supplementary material for: From Crystals to Disordered Crystals: A Hidden Order-Disorder Transition
Source: Sci Rep. 2015 Oct 20;5:15378. doi: 10.1038/srep15378 (PMC4613360; doi:10.1038/srep15378)
Supplement: Supplementary Information [file srep15378-s1.pdf]

Supplementary Information for "From Crystals to Disordered Crystals: A Hidden Order-Disorder Transition"

Hua Tong[1], Peng Tan[2], and Ning Xu[1,*]
[1]*CAS Key Laboratory of Soft Matter Chemistry, Hefei National Laboratory for Physical Sciences at the Microscale, and Department of Physics, University of Science and Technology of China, Hefei 230026, People's Republic of China*
[2]*State Key Laboratory of Surface Physics and Department of Physics, Fudan University, Shanghai 200433, People's Republic of China*
[*]*ningxu@ustc.edu.cn*

PART ONE: FURTHER ANALYSIS IN TWO DIMENSIONS (2D)

Section I. Structural amorphisation from disordered crystals to amorphous solids

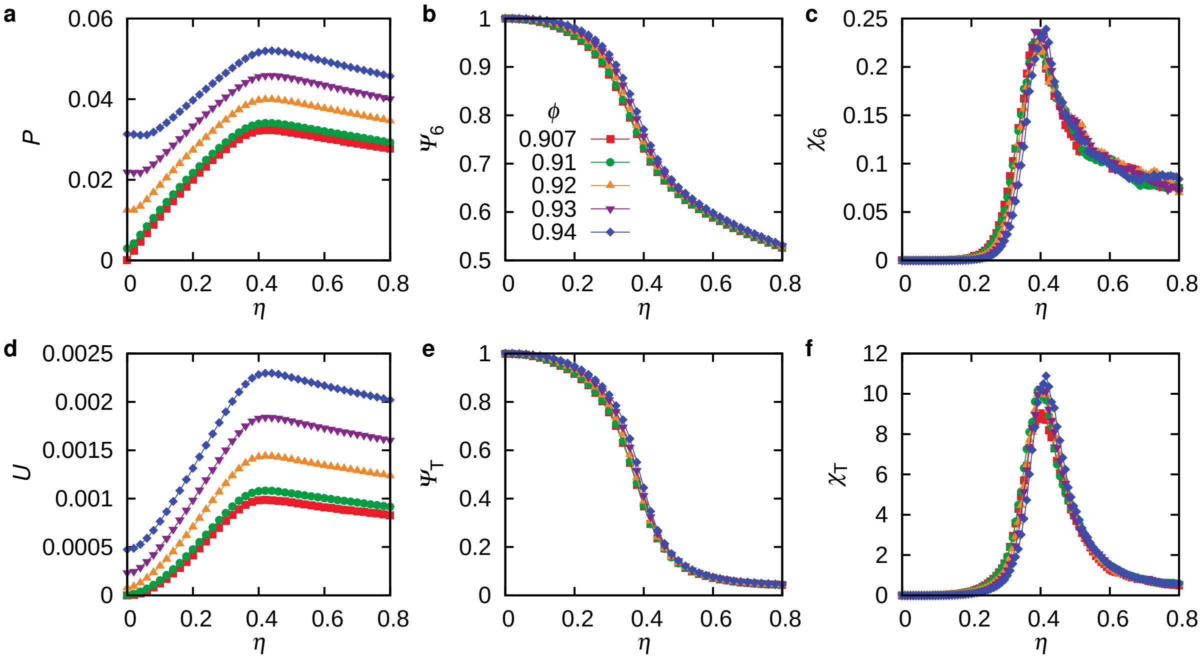

FIG. S1: **Polydispersity evolution of multiple quantities.** Polydispersity dependence of (**a**) pressure P, (**b**) bond orientational order parameter Ψ_6, (**c**) susceptibility of the bond orientational order χ_6, (**d**) potential energy per particle U, (**e**) translational order parameter Ψ_T, and (**f**) susceptibility of the translational order χ_T. Both P and U show a peak when the system undergoes the structural failure. At the same point, Ψ_6 and Ψ_T show a fast decay and their susceptibilities also exhibit a peak. The amorphisation transition signaled by the peak in χ_6 is shown by the squares in Fig. 1a of the main text.

As shown in the main text, a crystal undergoes a hidden order-disorder transition into the disordered crystal phase at a very small particle-size polydispersity. In this Section, we focus on the order-disorder transition at a sufficiently large polydispersity labeled by the squares in Fig. 1a of the main text, namely the structural amorphisation [1, 2].

Here we calculate two widely used geometric order parameters, the bond orientational order and translational order [3, 4]. The bond orientational order of particle j is given by

$$\Psi_{6j} = \left| \frac{1}{n_j} \sum_{k=1}^{n_j} e^{6i\theta_{jk}} \right|, \tag{1}$$

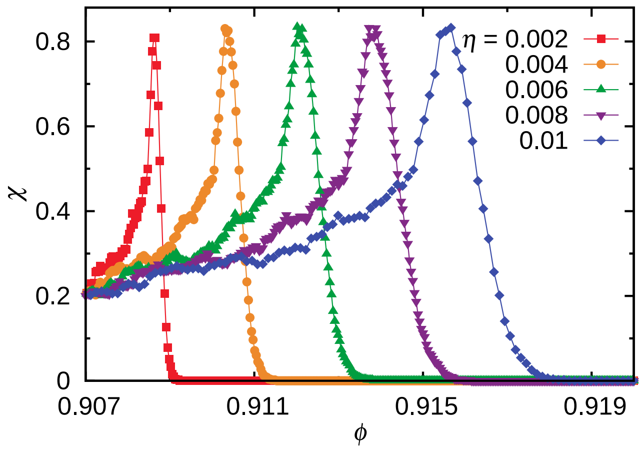

FIG. S2: **Order parameter susceptibility on the route of decompression.** Susceptibility of the order parameter χ as a function of the packing fraction ϕ obtained by decompressing the configurations from $\phi = 0.92$ at fixed polydispersities η. The transition points signaled by the peaks in $\chi(\phi)$ lie perfectly on the transition line obtained on the route of varying η at constant ϕ.

where n_j is the number of nearest neighbors of particle j, and θ_{jk} is the angle of the bond between particle j and its neighbor k with respect to the x axis. The global bond orientational order is the average over all particles: $\Psi_6 = \frac{1}{N}\sum_{i=1}^{N}\Psi_{6i}$. The translational order is defined as

$$\Psi_T = \frac{1}{N}\left|\sum_{j=1}^{N} e^{i\mathbf{G}\cdot\mathbf{r}_j}\right|, \tag{2}$$

where $\mathbf{G}$ is any first shell reciprocal lattice vector of a hexagonally close packed system. For a perfect hexagonal lattice, both Ψ_6 and Ψ_T are equal to one, while they decrease to small values for highly disordered solids. In order to unambiguously determine the amorphisation transition point, we calculate the order parameter susceptibilities

$$\chi_{6,T} = N(\langle\Psi_{6,T}^2\rangle - \langle\Psi_{6,T}\rangle^2), \tag{3}$$

where $\langle.\rangle$ denotes the average over 1000 realizations under the same macroscopic conditions.

Figure S1 shows the polydispersity evolution of the pressure P, potential energy per particle U, order parameters Ψ_6 and Ψ_T, and susceptibilities χ_6 and χ_T calculated at various packing fractions. With increasing the polydispersity η, P and U reach their peak values at $\eta_a \approx 0.4$. Meanwhile, both Ψ_6 and Ψ_T show a fast decay. The behaviors of Ψ_6 and Ψ_T across η_a resemble that of the two-dimensional melting [4]. As demonstrated by Figs. S1c and S1f, the transition points located by the peaks in $\chi_6(\eta)$ and $\chi_T(\eta)$ are identical, which signal a one-step transition from disordered crystals to amorphous solids. The transition of a (disordered) crystal into a glass, driven by quenched disorder, has recently been observed in experimental systems of colloidal suspensions [5].

Note that the critical packing fraction η_a of the amorphisation transition is almost independent of the packing fraction, in contrast to the linear scaling of the transition from crystals to disordered crystals with respect to the packing fraction reported in the main text. Moreover, Figs. S1b and S1e show that the geometric order parameters as a function of the polydispersity are also almost independent of the packing fraction, which are important evidences supporting the generality of Fig. 1a of the main text. If the polydispersity is replaced with either of the geometric order parameters or even other structural order parameters, Fig. 1a of the main text is still a valid phase diagram.

Section II. Alternate route probing the transition from crystals to disordered crystals

To demonstrate that the hidden order-disorder transition reported in the main text is robust and independent of the route, we verify in this section that the same transition occurs on the route of decompression at fixed particle-size polydispersity. In Fig. 1a of the main text, this route is perpendicular to that of varying the polydispersity

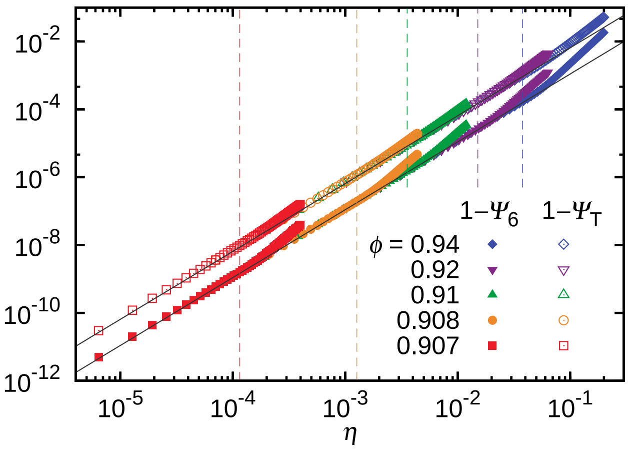

FIG. S3: **Geometric order parameters across the transition from crystals to disordered crystals.** Over a wide range of packing fractions, the deviations from perfect crystalline order of bond orientational order parameter $1-\Psi_6$ (filled points) and transitional order parameters $1-\Psi_T$ (open points) are plotted as functions of η. The vertical dashed lines label the critical polydispersities of the transition. The solid lines have a slope of 2, indicating the power law scalings in the crystal regime.

at fixed packing fraction to obtain the transition line labeled by the circles. We start from crystal states at high packing fractions and decompress them at fixed polydispersity by a small decrement of the packing fraction $\Delta\phi \in [3.625\times10^{-5}, 2\times10^{-4}]$, with smaller $\Delta\phi$ for smaller polydispersity. Potential energy minimization is performed after each decompression step. The packing fraction evolution of the order parameter susceptibility χ defined in the main text is shown in Fig. S2. There is also a peak in $\chi(\phi)$, signaling the hidden order-disorder transition. We have verified (not shown) that the transition points obtained here lie perfectly on the transition line (circles) shown in Fig. 1a of the main text.

Section III. Signs from the geometric order parameters across the hidden order-disorder transition

Seen from Figs. S1b and S1e, there is no observable sign of the transition from crystals to disordered crystals. However, we still expect to see some changes of the geometric order parameters across the transition. The idea is that the mechanical network breaks through the transition, which results in the change of the elastic properties (e.g., the bulk modulus B and shear modulus G shown in Figs. 2e-h of the main text) and should also be reflected in the geometric response. We then plot $1-\Psi_6$ and $1-\Psi_T$ as a function of the polydispersity and focus on the vicinity of the transition in Fig. S3. In the regime of crystals, both $1-\Psi_6$ and $1-\Psi_T$ are scaled well with η^2. Weak deviations from the power-law scalings can be observed after the crystals transit to disordered crystals.

Section IV. Another signature of the transition from crystals to disordered crystals from the mode analysis

Normal modes of vibration are the fundamentals to understanding properties of solids. In a finite-size crystal, the vibrational density of states $D(\omega)$ is composed of a set of δ-functions, with each δ-function containing degenerate modes. In a weakly disordered crystal, the mode degeneracy is broken and the δ-functions are broadened with a finite width positively correlated with the strength of disorder. Therefore, only in the presence of sufficiently strong disorder and/or for sufficiently large systems, $D(\omega)$ can be smoothed out. Constrained by the computational power, we are unable to study large enough systems to reliably resolve the boson peak at small polydispersities right above the transition from crystals to disordered crystals. Here we introduce an alternate characterization of the mode evolution with increasing the polydispersity η, which bypasses the issue of finite-size effect but shows an interesting and robust feature of the transition from the perspective of vibrational modes.

We directly trace the evolution of the mode frequencies in reference with those of a perfect crystal, which is

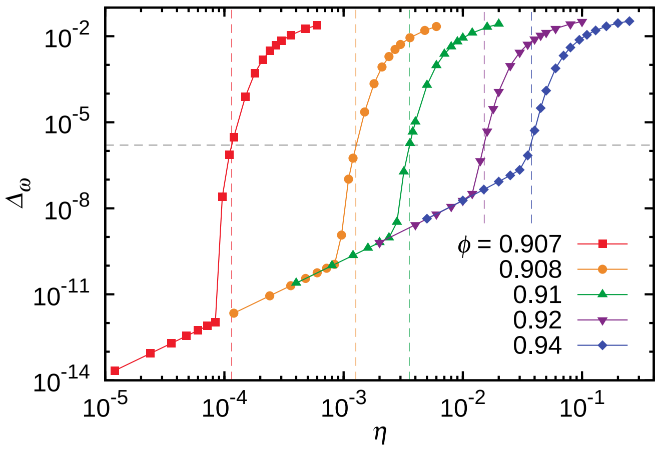

FIG. S4: **Mode evolution in reference to the perfect hexagonal crystal.** The departure of the vibrational modes from those of the perfect crystal Δ_ω as a function of the polydispersity η. The vertical dashed lines label the critical polydispersities η_c of the transition from crystals to disordered crystals. Interestingly, the critical Δ_ω at η_c are constant in packing fractions, as illustrated by the horizontal dashed line.

calculated as

$$\Delta_\omega = \left\langle \frac{1}{dN-d} \sum_{i=1}^{dN-d} \left(\frac{\omega_i - \omega_i^c}{\omega_i^c} \right)^2 \right\rangle, \tag{4}$$

where d is the dimension of space, ω_i and ω_i^c are the eigenfrequencies of the i-th mode of the solid with given polydispersity and of the perfect crystal, and $\langle . \rangle$ denotes the ensemble average. Due to the periodic boundary conditions, there are d zero-frequency modes, so the total number of nontrivial modes is $dN - d$. All the frequencies are sorted in the ascending order. At sufficiently small η, the i-th mode of a slightly deformed crystal only slightly deviates from the i-th mode of the perfect crystal, so that Δ_ω is an exact evaluation of the mode deviation from a perfect crystal. At larger η when mode interchanges take place, the i-th mode of the disordered solid may not be directly evolved from the i-th mode of the perfect crystal any more, so the expected one-to-one correspondence breaks. However, we still expect Δ_ω to be a good quantitative calculation of the deviation from perfect crystals. As shown in Fig. S4, Δ_ω increases with η. In the crystal regime, $\Delta_\omega \sim \eta^2$. Near the transition from crystals to disordered crystals defined by the peak in the order parameter susceptibility, Δ_ω grows abruptly. Interestingly, Δ_ω at the transition is independent of the packing fraction, as shown by the intersections between the data curves and vertical lines illustrated by the horizontal line in Fig. S4. This result is suggestive of a universal feature of the transition from crystals to disordered crystals. In combination with the observation that the transition at η_c may also signal the emergence of the boson peak, $\Delta_\omega \approx 1.6 \times 10^{-6}$ at η_c then sets a critical amount of deviation from a perfect crystal, above which the boson peak starts to appear. However, the origin of this particular value of Δ_ω is unknown at the moment, which deserves further investigations.

Section V. Finite size analysis of the transition from crystals to disordered crystals

In order to reveal the nature of a transition, finite size scaling is an important approach. As shown in Fig. S5, the critical polydispersity of N-particle systems satisfies the scaling $\eta_{c,N}(\phi) = \eta_{c,\infty}(\phi) + a(\phi)N^{-0.32}$, where $\eta_{c,\infty}(\phi)$ and $a(\phi)$ are packing fraction dependent constants. This scaling implies a diverging length $\xi \sim (\eta - \eta_c)^{-1.56}$. Meanwhile, the peak value of the susceptibility evolves very slowly with the system size, from which we resolve a very rough scaling relation: $\chi_{c,N}(\phi) = b(\phi)N^{0.044}$. To our knowledge, these scalings cannot be simply classified to well-known universalities of phase transitions. At current stage, it is not so easy to definitely determine the nature of the transition, like what people are facing to the jamming transition at zero temperature and shear stress. Intensive and careful studies are necessary to reveal the nature of the transition, which is one of our main goals in follow-up studies.

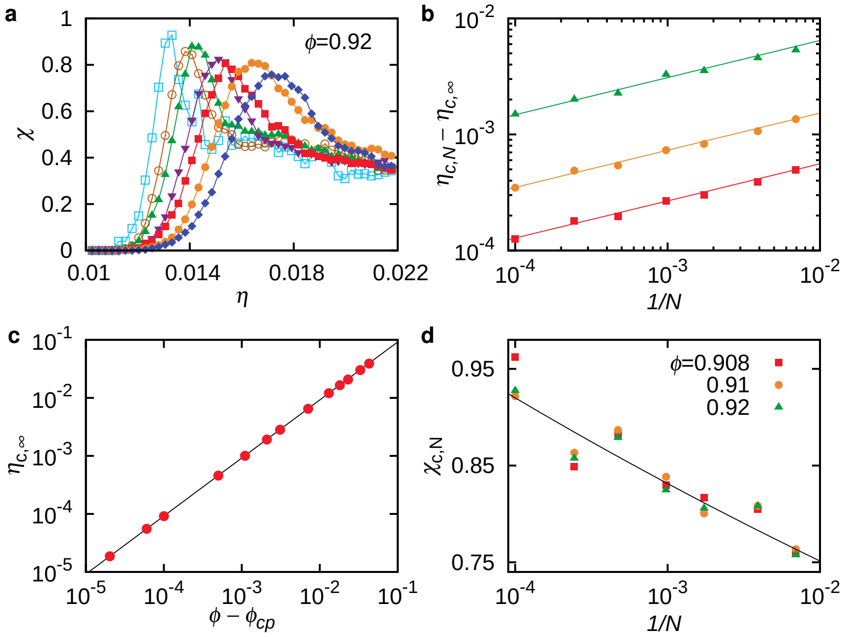

FIG. S5: **Finite size analysis of the transition from crystals to disordered crystals.** **a**, Susceptibility of the order parameter χ for different system sizes at $\phi = 0.92$. From the left to the right, N = 10000, 4096, 2116, 1024, 576, 256, and 144. **b**, System size dependence of the critical polydispersity $\eta_{c,N}$. The lines are the fittings to $\eta_{c,N} - \eta_{c,\infty} \sim N^{-0.32}$. **c**, Packing fraction dependence of $\eta_{c,\infty}$. The line is the fitting to $\eta_{c,\infty} \sim \phi - \phi_{\rm cp}$. **d**, System size dependence of the peak value of susceptibility of order parameter $\chi_{c,\infty}$. The solid line is the fitting to $\chi_{c,N} \sim N^{0.044}$. The packing fractions of the symbols in **b** and **d** are labeled in the legend of **d**.

Section VI. The transition from crystals to disordered crystals at finite temperatures

In this section, we show our preliminary results of the transition from crystals to disordered crystals at finite temperatures. Figure S6a shows the susceptibility of the fluctuation of the coordination number χ as a function of polydispersity η at $\phi = 0.92$ for different temperatures T. When increasing the temperature, the peak in $\chi(\eta)$ shifts to smaller η. This indicates that the thermal fluctuation is another factor in addition to the polydisopersity to induce frustrations. Figure S6b quantitatively shows that the critical polydispersity η_c [where $\chi(\eta_c)$ is maximal] decreases continuously from its zero temperature value with increasing temperature. Therefore, our zero temperature results are not singular from those of thermal systems, but are rather the low temperature limit. Figure S6c shows how the crystal-disordered crystal transition line evolves with temperature. The temperature evolution is apparently continuous, so the phenomenology that we reported for zero temperature systems does not change in thermal systems and is representative.

PART TWO: ANALYSIS IN THREE DIMENSIONS (3D)

In the main text and PART ONE of this Supplementary Information, we have shown that a hidden order-disorder transition from crystals to disordered crystals occurs at a rather small polydispersity, followed by the structural amorphisation at sufficiently large polydispersity in 2D. Here we perform extensive studies in 3D and confirm that the results are essentially the same. The model has been described in Methods of the main text.

Section I. Order parameter and elastic properties across the transition from crystals to disordered crystals

Figures S7a-c and S7e-h show the η evolution of the average coordination number z, order parameter δz, order parameter susceptibility χ, bulk and shear moduli B and G, and nonaffinity upon compression and shear μ_c and μ_s. All quantities undergo significant changes across the critical polydispersity η_c, which is defined from the first sharp

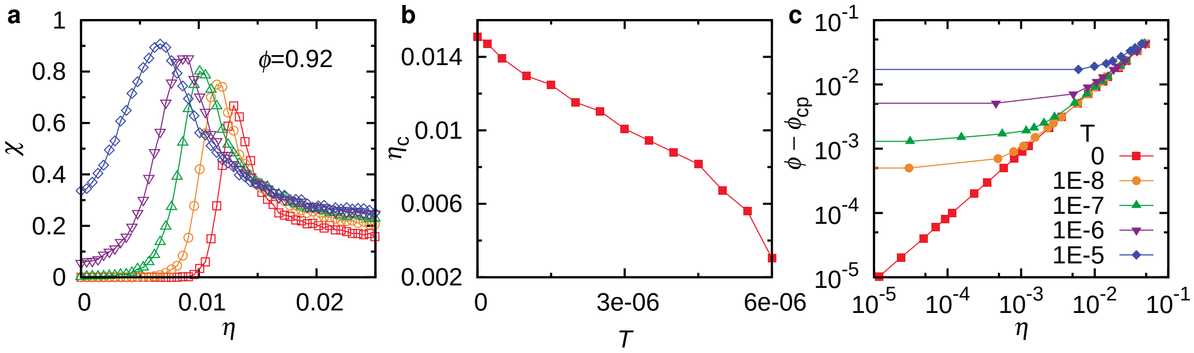

FIG. S6: **Transition from crystals to disordered crystals at finite temperatures. a**, Polydispersity evolution of the susceptibility of the order parameter χ at $\phi = 0.92$ for different temperatures. From left to right, $T = 5 \times 10^{-6}$, 4×10^{-6}, 3×10^{-6}, 2×10^{-6}, and 10^{-6}. **b**, Critical polydispersity η_c as a function of the temperature at $\phi = 0.92$. **c**, Crystal-disordered crystal transition lines for different temperatures. The lines are to guide the eye.

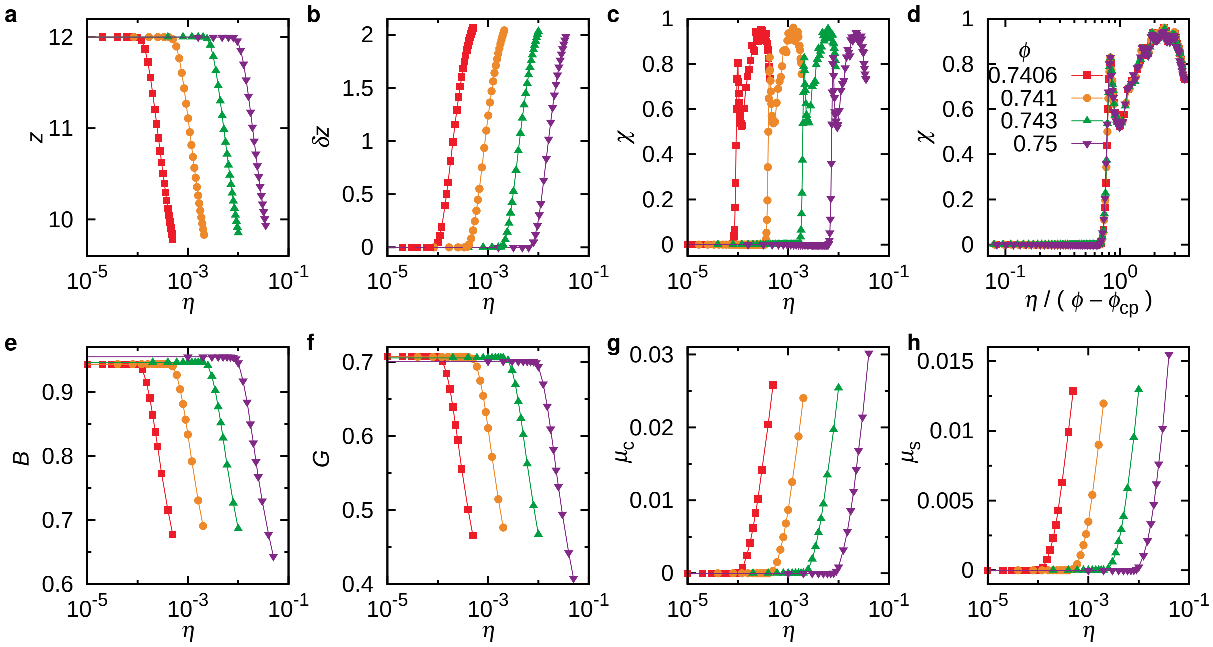

FIG. S7: **Order parameter and elastic properties across the transition from crystals to disordered crystals in 3D**. **a-c**, Polydispersity evolution of the average coordination number z, fluctuation of the coordination number δz as the order parameter, and susceptibility of the order parameter χ. The critical polydispersity of the transition η_c is determined by the location of the sharp peak (first one on the left) in $\chi(\eta)$. **d**, Scaling collapse of all curves in **c** when χ is plotted against $\eta/(\phi-\phi_{cp})$. **e-h**, Polydispersity evolution of the bulk modulus B, shear modulus G, nonaffinity of the compression deformation μ_c, and nonaffinity of the shear deformation μ_s.

peak (the one on the left) in $\chi(\eta)$ of Fig. S7c as the transition point from crystals to disordered crystals. As illustrated by circles in Fig. S11, $\eta_c \sim \phi - \phi_{cp}$, where $\phi_{cp} = \sqrt{2}\pi/6$ is the packing fraction of close-packed hard spheres in 3D. Therefore, the close packing point is also singular in 3D, where infinitesimally small polydispersity will trigger the crystal-disordered crystal transition.

It is worth noting that for a perfect crystal with face-centred cubic lattice, the original coordination number is $z = 12$, which is much larger than the isostatic value $z_{iso} = 6$. Note that the coordination number is defined as the average number of particles with which a given particle interacts, different from the number of neighbors determined from the Voronoi construction. Therefore, with the same amount of increase in polydispersity, much more contacts

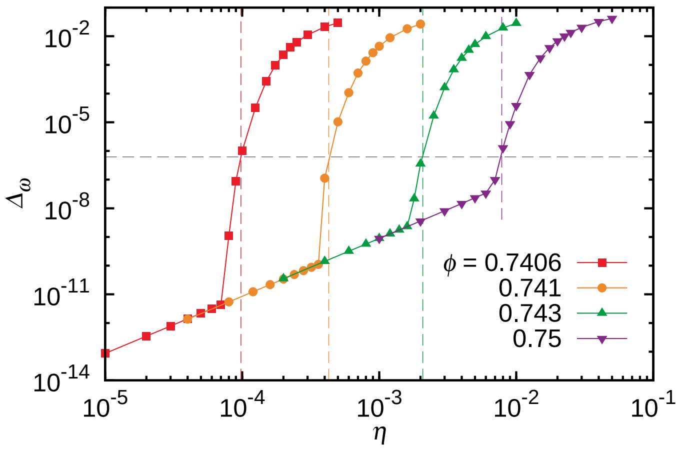

FIG. S8: **Mode evolution in reference to the perfect face-centred cubic crystal.** The departure of the vibrational modes from those of the perfect crystal Δ_ω as a function of the polydispersity η. The vertical dashed lines label the critical polydispersities η_c of the transition from crystals to disordered crystals. Interestingly, the critical Δ_ω ($\approx 6.2 \times 10^{-7}$) at η_c are constant in packing fractions, as illustrated by the horizontal dashed line.

break in 3D than in 2D. As shown in Fig. S7a, a second contact breaks for each particle in average within the regime of polydispersity under investigation, while less than one contact breaks in 2D (see Fig. 2a of the main text). This is reflected by the second peak (the broader one on the right) in $\chi(\eta)$ of Fig. S7c. Interestingly, when we plot χ against $\eta/(\phi - \phi_{\rm cp})$ in Fig. S7d, all curves collapse nicely onto the same master curve, including the high-η part. This confirms $\eta/(\phi - \phi_{\rm cp})$ as a more meaningful parameter in control of properties of solids close to the crystal-disordered crystal transition, both in 2D and 3D.

Section II. Signature of the transition from crystals to disordered crystals from the mode analysis

As discussed in Section IV of PART ONE, finite system size inevitably affects the calculation of the density of vibrational states $D(\omega)$. In 3D, we are more severely constrained by the computational power to resolve the boson peak with a reliable precision. This difficulty hinders a quantitative study of the relation between the formation of the boson peak and the crystal-disordered crystal transition, as done in 2D. Our preliminary results indicate that the boson peak should still emerge around η_c.

Here we employ the quantity Δ_ω introduced in equation (4) to characterize the hidden order-disordered transition from the perspective of vibrational modes. As shown in Fig. S8, Δ_ω increases abruptly near the transition from crystals to disordered crystals and its value at the transition is independent of the packing fraction. This result further suggests that Δ_ω is a good quantitative calculation of the deviation from a perfect crystal, which unveils a universal feature of the transition from crystals to disordered crystals.

Section III. Scaling analysis across the transition from crystals to disordered crystals

In this section, we demonstrate how the physics of crystals evolves to the physics of jamming in 3D. We start from a static packing at $\phi = 0.75$ and quasistatically decrease the packing fraction at fixed polydispersity to the desired pressure. Figure S9 shows the pressure dependence of G/B and $z - z_{\rm iso}$ at different η, spanning on both sides of the hidden order-disorder transition and with amorphous solids at $\eta = 0.6$ as a reference. As expected, G/B and $z - z_{\rm iso}$ stay constant in the crystal regime and recover the jamming scalings away from the hidden order-disorder transition. For disordered crystals close to the transition, we observe unique pressure scaling apart from the physics of crystals and jamming: $G/B \sim P^{0.72}$ and $z - z_{\rm iso} \sim P^{0.85}$. Therefore, an intermediate family of solids complying physics other than those of crystal and jamming universally exists in both 2D and 3D which deserves further investigation.

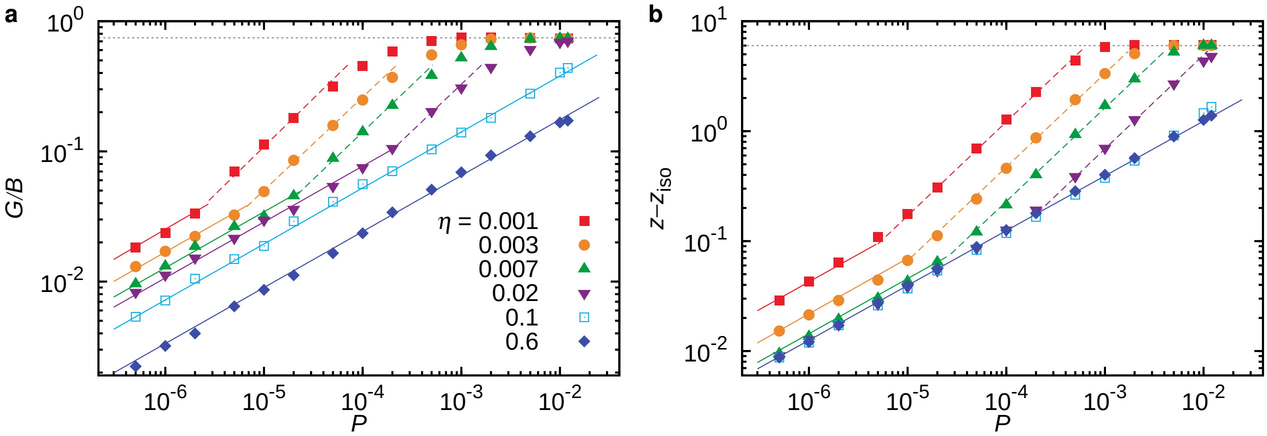

FIG. S9: **Scaling behaviors of the elastic moduli and coordination number in 3D. a-b**, Pressure evolution of the ratio of the shear modulus to the bulk modulus G/B and excess coordination number $z - z_{\rm iso}$. The horizontal dotted lines show the crystal behavior. The solid (dashed) lines are power-law fits to the data: $G/B \sim P^{0.43}$ ($G/B \sim P^{0.72}$) and $z - z_{\rm iso} \sim P^{0.5}$ ($z - z_{\rm iso} \sim P^{0.85}$). Both G/B and $z - z_{\rm iso}$ are independent of P in the crystal regime and recover the jamming scalings away from the transition from crystals to disordered crystals. Close to the transition, a third scaling behavior exists between those of crystalline and jammed packings.

Section IV. Structural amorphisation from disordered crystals to amorphous solids

Here we characterize the structural amorphisation in 3D. The bond orientational order of particle j is now given by

$$Q_{6i} = \sqrt{\frac{4\pi}{13}\sum_{m=-6}^{6}|Q_{6m,i}|^2}, \tag{5}$$

where $Q_{6m,i} = \frac{1}{n_i}\sum_{j=1}^{n_i} Y_{6m}(\vec{r_{ij}})$ with n_i the number of nearest neighbors of particle i and $Y_{6m}(\vec{r_{ij}})$ the spherical harmonics. The global bond orientational order is the average over all particles: $Q_6 = \frac{1}{N}\sum_{i=1}^{N} Q_{6i}$. The transitional order is defined the same as equation (2) but with $\mathbf{G}$ the first shell reciprocal lattice vector of a close packing with face-centred cubic structure. The susceptibilities of Q_6 and Ψ_T are calculated according to equation (3) to unambiguously determine the amorphisation transition point.

Figure S10 shows the η evolution of the pressure P, potential energy per particle U, order parameters Q_6 and Ψ_T and their susceptibilities χ_6 and χ_T for various packing fractions. The amorphisation transition is signaled by the peak values of P and U, and also a fast decay of both Q_6 and Ψ_T. We determine the transition points from the peaks of order parameter susceptibility $\chi_6(\eta)$ and $\chi_T(\eta)$, which are illustrated by squares in Fig. S11 of the phase diagram.

Section V. Phase diagram

A unified phase diagram of solids in 3D over the entire spectrum of disorder is conveyed in Fig. S11 with the hidden order-disorder transition from crystals to disordered crystals and the structural amorphisation from disordered crystals to amorphous solids. The similarity between the phase diagrams in 2D and 3D confirms their generality. We hope that they can help to rationalize our understanding of solids in various regions of disorder.

PART THREE: ANALYSIS FOR 2D SYSTEMS WITH HERTZIAN REPULSION AND GAUSSIAN PARTICLE-SIZE DISTRIBUTION

In the main text and PART TWO of this Supplementary Information, we have shown that a hidden order-disorder transition from crystals to disordered crystals occurs for systems with harmonic repulsion and a uniform particle-size distribution in both 2D and 3D. Here we perform extensive studies in 2D systems with Hertzian repulsion and Gaussian particle-size distribution to confirm that the physics associated with the transition are not limited to the

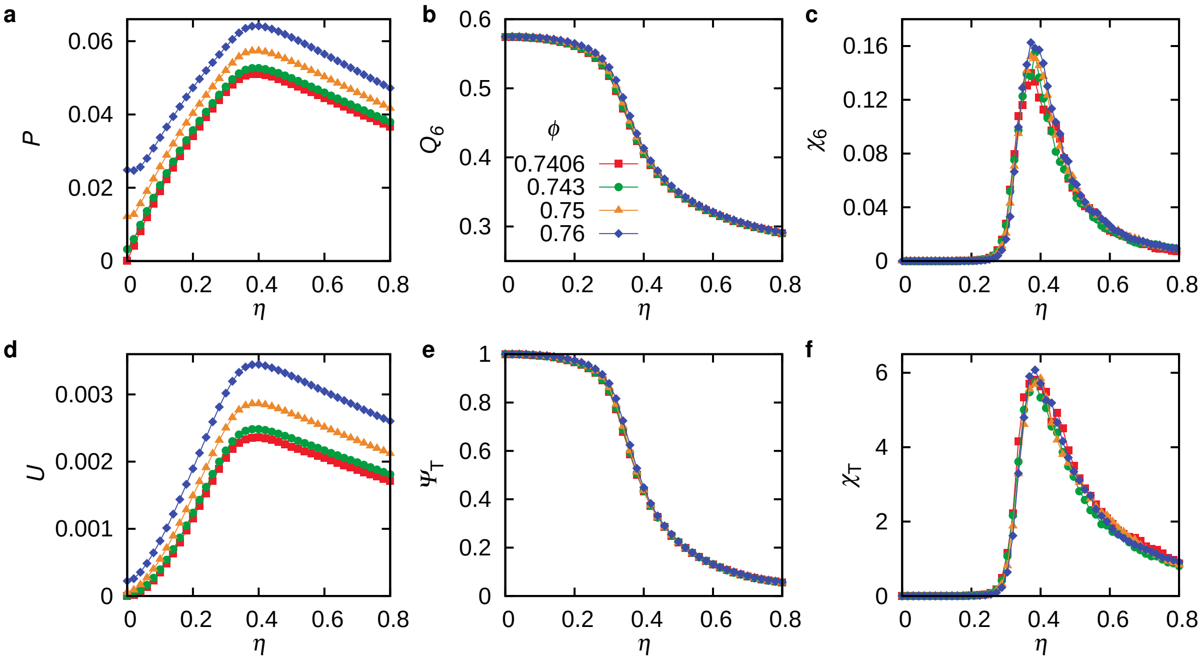

FIG. S10: **Polydispersity evolution of multiple quantities in 3D.** Polydispersity dependence of (**a**) pressure P, (**b**) bond orientational order parameter Q_6, (**c**) susceptibility of the bond orientational order χ_6, (**d**) potential energy per particle U, (**e**) translational order parameter Ψ_T, and (**f**) susceptibility of the translational order χ_T. Both P and U show a peak when the system undergoes the structural failure. At the same point, Q_6 and Ψ_T show a fast decay and their susceptibilities also exhibit a peak.

specific model discussed in the main text of the paper. The polydispersity is now defined as $\eta = \sqrt{\langle\sigma^2\rangle - \langle\sigma\rangle^2}/\langle\sigma\rangle$, where the bracket denotes average over all particles.

Section I. Order parameter and elastic properties across the transition from crystals to disordered crystals

With increasing polydispersity, there are three sources of disorder: (1) the geometric distortion from perfect crystalline lattice, (2) the spatial variation of the coordination number, and (3) the spatial variation of the effective"spring constant" of particle interactions. The first one is negligible because the transition happens at a small polydispersity with rather high crystalline order. In the main text, we stick to harmonic repulsion because the third one is also absent, so that we can construct the order parameter purely from the fluctuation of the coordination number, which precisely characterizes the strength of disorder. For Hertzian repulsion, the spatial fluctuation of the effective "spring constant" is inevitable when polydispersity is present and may work together with the coordination number to quantitatively determine the transition from crystals to disordered crystals.

Figures S12d-f show the polydispersity evolutions of the average spring constant $K = \langle k\rangle$, its fluctuation $\delta K = \sqrt{\langle k^2\rangle - \langle k\rangle^2}$, and relative fluctuation $\delta K/K$, where k is the spring constant of a single bond and the bracket denotes the average over all bonds. Here $\delta K/K$ (analogous with δz) quantifies the disorder induced by the variation of spring constant. Compared to the "sharp" changes in the coordination number related quantities across the transition seen in Fig. S12a-c, the evolutions of spring constant related quantities are much more gentle.

Ideally, an order parameter taking into account the contributions from fluctuations of both the coordination number and spring constant can be constructed to characterize the transition. At current stage, however, we are not clear how to construct such an order parameter, which may be an interesting issue to tackle in future studies. seen from Figs. S12-S15, δz is still a pretty decent order parameter to characterize the transition for Hertzian repulsion. Compared to the coordination number, the fluctuation of the spring constant also monotonically increases with polydispersity, but in a much smoother way across the transition. Therefore, similar to the harmonic case, the fluctuation of the coordination number dominates the transition. Furthermore, the bottom panels of Fig. S12 also shows that near the transition K reaches the minimum and $\delta K/K$ exhibits a fast increase, which should also result from the qualitative change of

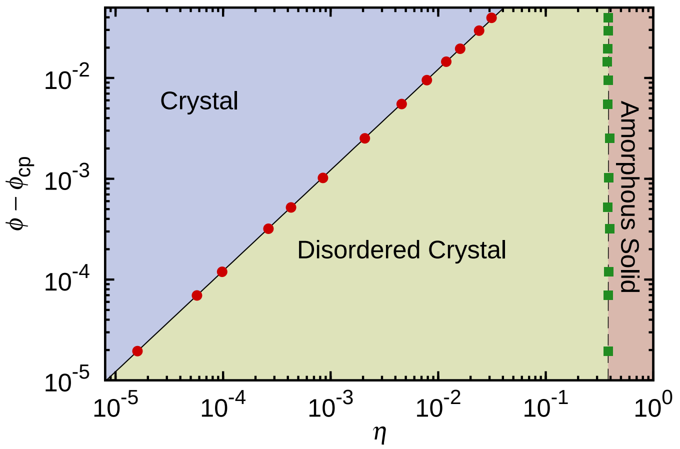

FIG. S11: **Phase diagram in 3D**. Phase diagram with two order-disorder transitions in the parametric space of the particle-size polydispersity η and packing fraction distance from the close packing of hard spheres $\phi - \phi_{\rm cp}$. With increasing η at fixed ϕ, the system starting from a perfect face-centred cubic crystal undergoes a transition at η_c toward a disordered crystal state, which is labeled by the circles with the linear fit $\eta_c \sim \phi - \phi_{\rm cp}$ (solid line). $\eta_c = 0$ when $\phi = \phi_{\rm cp}$, indicating that the close packing point is singular. Across this transition, the geometric structure maintains an extremely high crystalline order, whereas mechanical and vibrational properties become more like disordered solids. The transition labeled by the squares signals the structural amorphisation from disordered crystals to amorphous solids. The dashed line is to guide the eye.

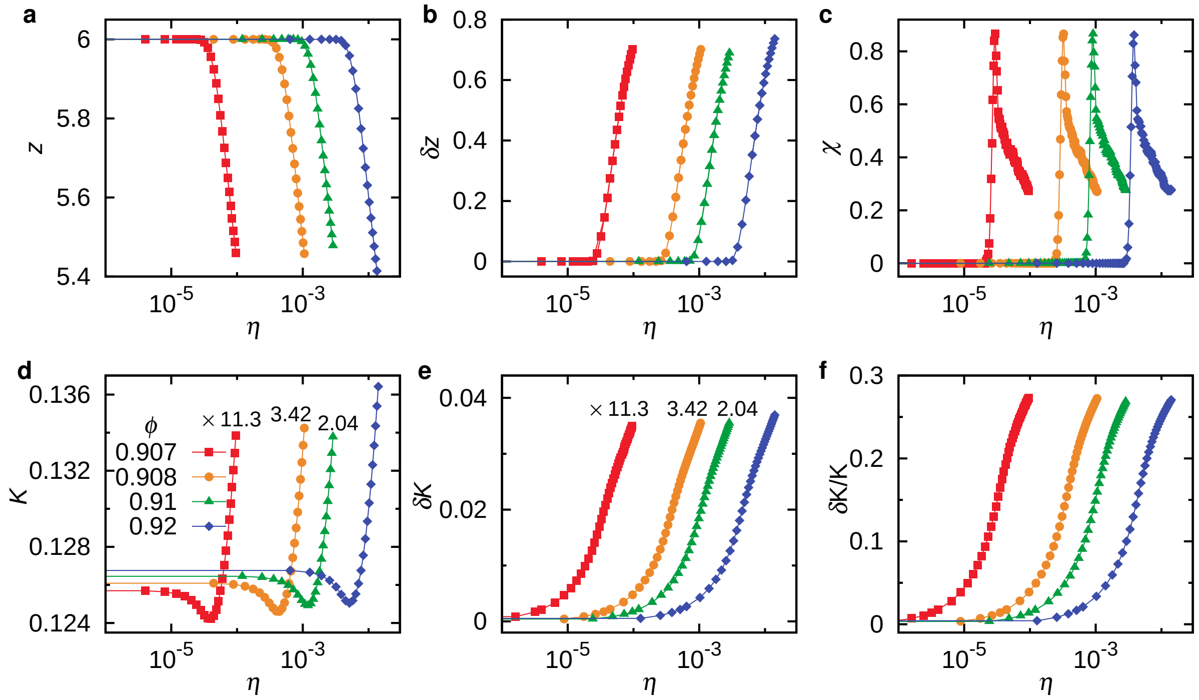

FIG. S12: **Coordination number and spring constant related quantities across the transition from crystals to disordered crystals for 2D systems with Hertzian repulsion**. **a-c**, Polydispersity evolution of the average coordination number z, its fluctuation δz as the order parameter, and susceptibility of the order parameter χ. **d-f**, Polydispersity evolutions of the average spring constant K, its fluctuation δK, and the relative fluctuation $\delta K/K$.

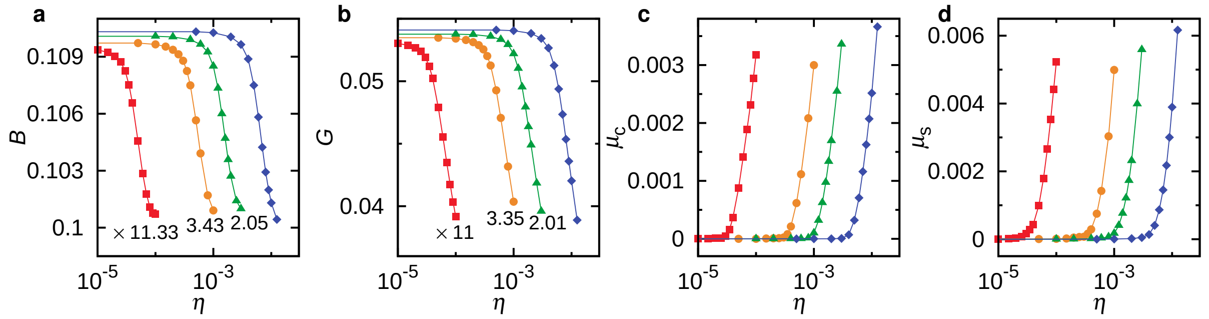

FIG. S13: **Elastic properties across the transition from crystals to disordered crystals for 2D systems with Hertzian repulsion. a-d** Polydispersity evolutions of the bulk modulus B, shear modulus G, and nonaffinities upon compression μ_c and shear μ_s. Symbols are the same as in Fig. S12.

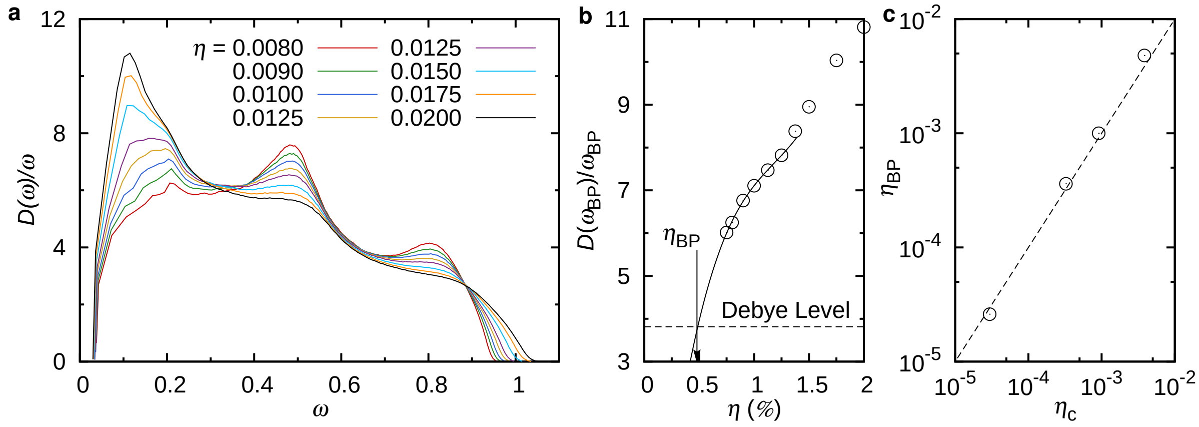

FIG. S14: **Formation of the boson peak in 2D systems with Hertzian repulsion. a**, Reduced density of vibrational states $D(\omega)/\omega$ at $\phi = 0.92$ and different polydispersities. **b**, Polydispersity evolution of the strength of the boson peak $D(\omega_{\rm BP})/\omega_{\rm BP}$ from **a**. The solid line is a polynomial fit to the low η data: $y = -5.07 + 2678.83x - 203217.90x^2 + 5710223.57x^3$. It hits the Debye level labeled by the horizontal dashed line at $\eta_{\rm BP}$. **c**, Correlation between $\eta_{\rm BP}$ and η_c. The data points correspond to five packing fractions of $\phi = 0.907$, 0.908, 0.91, and 0.92 in the ascendent order of η_c. The dashed line shows $\eta_{\rm BP} = \eta_c$.

the mechanical network signaled by the fluctuation of the coordination number. Therefore, in the Hertzian case, the fluctuation of the spring constant does not determine the transition, whereas its behaviors are coupled to and affected by those of the coordination number.

Figure S13 shows the η evolutions of the elastic moduli and nonaffinities upon deformation. Due to the evolution of averaged spring constant as shown in Fig. S12d, B and G gently decrease before the fast decay across the transition. However, the evolutions of the nonaffinities shown in Figs. S13c and 13d still exhibit a "sharp" increase across the critical polydispersity η_c, as seen for harmonic repulsion. The nonaffinity is the right quantity to characterize disordered systems, so its behavior is more representative than elastic moduli to characterize the transition.

Section II. Signature of the transition from crystals to disordered crystals from the mode analysis

Figure S14a shows the reduced density of states $D(\omega)/\omega$ for disordered crystals at $\phi = 0.92$. With increasing η, the boson peak (the first peak at low frequencies) gradually rises and moves to lower frequencies, as seen for harmonic repulsion. $D(\omega_{\rm BP})/\omega_{\rm BP}$ against η is plotted in Fig. S14b with $\omega_{\rm BP}$ the boson peak frequency. Due to the nonlinear nature of Hertzian replusion, the low-η data apparently deviates from the linear behavior as in the harmonic case. We then empoly the polynomial fit to estimate the evolution of the boson peak. As shown in Fig. S14b, the fitting is resonable, which hits the Debye level at $\eta_{\rm BP} \approx \eta_c$. Figure S14c shows that $\eta_{\rm BP} \approx \eta_c$ over a wide range of packing

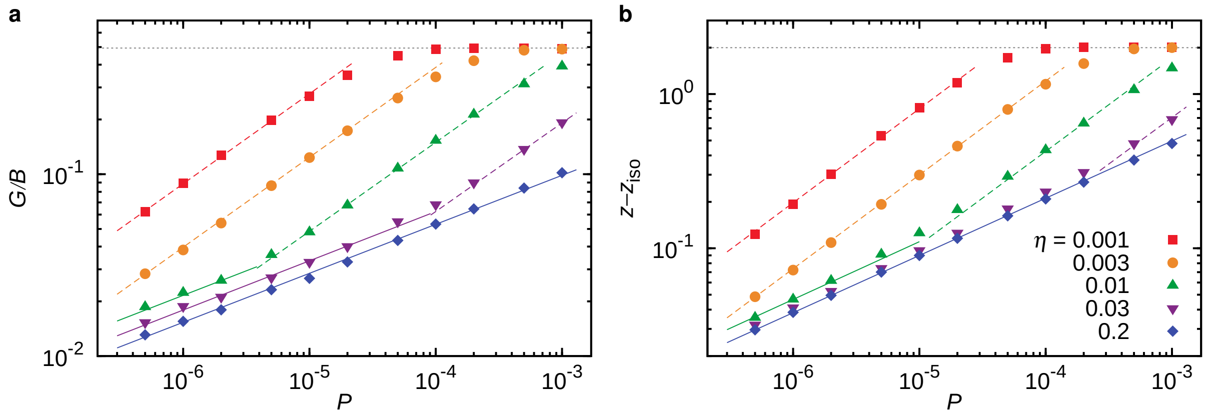

FIG. S15: **Scaling behaviors of the elastic moduli and coordination number in 2D systems with Hertzian repulsion.** **a-b**, Pressure evolution of the ratio of the shear modulus to the bulk modulus G/B and excess coordination number $z - z_{\rm iso}$. The horizontal dotted lines show the crystal behavior. The solid (dashed) lines are power-law fits to the data: $G/B \sim P^{0.27}$ ($G/B \sim P^{0.48}$) and $z - z_{\rm iso} \sim P^{0.37}$ ($z - z_{\rm iso} \sim P^{0.61}$).

fractions from $\phi = 0.907$ to 0.92. Although the small deviation between $\eta_{\rm BP}$ and η_c may indicate that a more precise order parameter is needed to determine the exact location of the transition and a more suitable fitting function is needed to predict the evolution of the boson peak, the evidence is clear that the formation of the boson peak is correlated with the transition from crystals to disordered crystals.

Section III. Scaling analysis across the transition from crystals to disordered crystals

In this section, we demonstrate how the physics of crystals evolves to the physics of jamming in 2D systems with Hertzian repulsion and Gaussian particle-size distribution. Figure S15 shows the pressure dependence of G/B and $z - z_{\rm iso}$ at different η, spanning on both sides of the hidden order-disorder transition and with amorphous solids at $\eta = 0.2$ as a reference. As expected, G/B and $z - z_{\rm iso}$ stay constant in the crystal regime and recover the jamming scalings away from the hidden order-disorder transition. For disordered crystals close to the transition, we observe unique pressure scaling apart from the physics of crystals and jamming: $G/B \sim P^{0.48}$ and $z - z_{\rm iso} \sim P^{0.61}$. Therefore, the existence of an intermediate family of solids complying physics other than those of crystal and jamming is universal for both harmonic and Hertzian repulsions and is independent of the style of the particle-size polydispersity.

[1] Mizuno, H., Mossy, S. & Barrat, J.-L. Elastic heterogeneity, vibrational states, and thermal conductivity across an amorphisation transition. *Europhys. Lett.* **104**, 56001 (2013).

[2] Mizuno, H., Mossa, S. & Barrat, J.-L. Acoustic excitations and elastic heterogeneities in disordered solids. *Proc. Natl Acad. Sci. USA* **111**, 11949 (2014).

[3] Steinhardt, P. J., Nelson, D. R. & Ronchetti, M. Bond-orientational order in liquids and glasses. *Phys. Rev. B* **28**, 784 (1983).

[4] Han, Y., Ha, N. Y., Alsayed, A. M. & Yodh, A. G. Melting of two-dimensional tunable-diameter colloidal crystals *Phys. Rev. E* **77**, 041406 (2008).

[5] Yunker, P., Zhang, Z. & Yodh, A. G. Observation of the disorder-induced crystal-to-glass transition. *Phys. Rev. Lett.* **104**, 015701 (2010).
